# The role of miR1 and miR133a in new-onset atrial fibrillation after acute myocardial infarction

**DOI:** 10.1186/s12872-023-03462-x

**Published:** 2023-09-11

**Authors:** Qingyi Zeng, Wei Li, Zhenghua Luo, Haiyan Zhou, Zhonggang Duan, Xin Lin Xiong

**Affiliations:** 1 Guizhou Medical University, 9 Beijing Road, Guiyang, 550000 Guizhou China; 2 The Second Affiliated Hospital of Guizhou University of Chinese Medicine, 83 Feishan Street, Guiyang, China; 3 Affiliated Hospital of Guizhou Medical University, 16 Beijing Road, Guiyang, 550000 Guizhou China; 4 Guizhou Provincial People’s Hospital, 83 Zhongshan East Road, Guiyang, 55000 Guizhou China

**Keywords:** New-onset atrial fibrillation, Acute myocardial infarction, miR1, miR133a, Inflammation, Glasgow prognostic score, Left atrial diameter, Blood sedimentation

## Abstract

**Background:**

The development of new-onset atrial fibrillation (NOAF) after acute myocardial infarction (AMI) is a clinical complication that requires a better understanding of the causative risk factors. This study aimed to explore the risk factors and the expression and function of miR-1 and miR-133a in new atrial fibrillation after AMI.

**Methods:**

We collected clinical data from 172 patients with AMI treated with emergency percutaneous coronary intervention (PCI) between October 2021 and October 2022. Independent predictors of NOAF were determined using binary logistic univariate and multivariate regression analyses. The predictive value of NOAF was assessed using the area under the receiver operating characteristic (ROC) curve for related risk factors. In total, 172 venous blood samples were collected preoperatively and on the first day postoperatively; the expression levels of miR-1 and miR-133a were determined using the polymerase chain reaction. The clinical significance of miR-1 and miR-133a expression levels was determined by Spearman correlation analysis.

**Results:**

The Glasgow prognostic score, left atrial diameter, and infarct area were significant independent risk factors for NOAF after AMI. We observed that the expression levels of miR-1 and miR-133a were significantly higher in the NOAF group than in the non-NOAF group. On postoperative day 1, strong associations were found between miR-133a expression levels and the neutrophil ratio and between miR-1 expression levels and an increased left atrial diameter.

**Conclusions:**

Our findings indicate that the mechanism of NOAF after AMI may include an inflammatory response associated with an increased miR-1-related mechanism. Conversely, miR-133a could play a protective role in this clinical condition.

## Background

Acute myocardial infarction (AMI) is the acute necrosis of coronary artery stenosis caused by corresponding myocardial ischemia. New-onset atrial fibrillation (NOAF) is a critical complication with an incidence rate of 6–22% [1]. Patients with NOAF have a 40% higher mortality rate than those with normal sinus rhythm [2]. Long-term follow-up results of relevant studies have shown a lower incidence of ischemic cerebrovascular disease or transient ischemic attacks in patients with non-valvular atrial fibrillation (NVAF) in the rhythm control group [3]. The specific mechanism of atrial fibrillation has not been defined but involves electrical and structural remodelling of the atrium. Structural remodelling includes increased atrial fibrosis, increasing ectopic electrical activity and conduction anisotropy within the atrial tissue [4, 5]. In patients with a mild reduction in ejection fraction (HFmrEF), a left atrial volume index (LAVI) > 30.5 can predict the presence of atrial fibrillation with a sensitivity of 64% and specificity of 66% [6]. However, electrical remodelling mainly involves changes in the expression of cardiac ion channels, leading to the shortening of the atrial action potential duration and effective refractory period and changes in atrial calcium homeostasis [7–10]. Previous studies have indicated that fragmented QRS (fQRS) is an independent determinant of atrial fibrillation in patients with ST-segment elevation myocardial infarction (STEMI) [11]. The P-wave peak times in V1 and D2 leads, which are obtained from surface electrocardiography (ECG), are highly predictive in determining the likelihood of patients developing atrial high-rate episodes (AHRE) [12].

MiRNAs are small noncoding RNAs that regulate gene and protein expression by causing mRNA degradation or translational repression [13]. Among these miRNAs, miR-1 and miR-133a are specifically expressed in adult heart and skeletal muscle tissues, and their expression patterns vary depending on the pathological condition [14]. This study aimed to investigate the risk factors for NOAF after AMI, assess the expression levels of miR-1 and miR-133a before and after AMI, and examine their potential clinical significance as risk factors for NOAF.

## Methods

### Study participants and specimen collection

Between October 2021 and October 2022, 172 patients with AMI who underwent emergency percutaneous coronary intervention (PCI) at the Affiliated Hospital of Guizhou Medical University were consecutively selected as participants for this study. The patients’ medical records were used to collect clinical data during hospitalisation. This data included various categorical and continuous variables such as age, gender, and biochemical indicators. In addition, the Glasgow Prognostic Score (GPS) was calculated to assess the inflammatory status and predict the prognosis of the patients. The GPS is determined by measuring two acute-phase proteins in the blood: C-reactive protein (CRP) and albumin.

Furthermore, the results of coronary angiography were also recorded for analysis. In this study, 172 blood samples were collected from the participants before undergoing PCI and on the first day after the intervention. These samples were used to analyse plasma miR1 and miR133a expression levels. The Ethics Committee of the Affiliated Hospital of Guizhou Medical University approved the study protocol, and the study was conducted following the principles outlined in the Declaration of Helsinki. Informed consent was obtained from all patients before collecting the samples.

### Inclusion and exclusion criteria

AMI diagnostic criteria refer to the Fourth Global Unified Definition of Myocardial Infarction, published in 2018 [15], where acute myocardial injury exists and at least one of the following conditions is present: (1) symptoms of a lack of blood perfusion in the myocardium or chest pain lasting for > 30 min; (2) newly developed ischemic electrocardiogram (ECG) changes; (3) the formation of a new pathological Q wave on ECG; (4) imaging evidence confirming the presence of newly inactivated myocardium or ventricular wall motion abnormalities; and (5) angiography confirming an intracoronary thrombosis or an autopsy. The ECG diagnostic criteria for atrial fibrillation were: disappearing P wave, replaced with fibrillation wave (f wave) of different sizes and shapes; frequency of the f wave of 350–600 times/min; and irregular R–R interval. NOAF was defined as no history of paroxysmal or persistent atrial fibrillation, atrial flutter, or the first episode of atrial fibrillation on admission or during hospitalisation. The duration of atrial fibrillation was assessed under any of the following conditions: (1) the duration of atrial fibrillation could be determined by a complete 12-lead ECG or recorded by a Holter monitor and (2) ECG monitoring of atrial fibrillation for at least 30 s [16, 17]. The following exclusion criteria were applied: (1) a history of heart disease; (2) known malignant tumours; (3) thyroid disease or severe liver and renal insufficiency; (4) recent surgery and trauma; (5) combined acute and chronic infections and recent cerebrovascular diseases; (6) autoimmune diseases or a history of skeletal muscle trauma and other diseases; (7) history of atrial flutter, myocardial infarction, myocarditis, or cardiomyopathy; (8) history of radiofrequency ablation for arrhythmia, coronary artery bypass grafting, or other cardiac surgeries; and (9) severe valvular lesions or congenital heart disease.

### Sample collection

Venous blood samples (5 mL) were collected from patients with AMI before PCI and on the first day after PCI using EDTA-K2 anticoagulant tubes. All blood samples were centrifuged at 4 °C (13,400×g, 10 min), and the plasma was transferred to RNase/DNA microcentrifuge tubes and stored at -80℃.

### Quantitative real-time polymerase chain reaction (qRT-PCR) of miR1 and miR133a

Total RNA was isolated using a total RNA extraction kit (Tiangen Biochemical Technology Company, Beijing, China), and miR1 and miR133a expression levels were quantified using the Bulge-Loop™ miRNA qRT-PCR starter kit (Ruibo Biotechnology, Guangzhou, China) with the following reverse transcription program: 42 °C for 60 min and inactivated reverse transcriptase at 70 °C for 10 min. After terminating the reaction, the resulting product was cooled on ice and stored at -80℃. The reverse transcription and PCR reaction system was configured as previously described, and PCR reactions were performed using a fluorescent PCR instrument (Bio-Rad Laboratories, Hercules, CA, USA). The PCR reaction conditions were as follows: pre-denaturation at 95 °C for 10 min, denaturation at 95℃ for 2 s, annealing at 60℃ for 30 s, and extension at 70℃ for 10 s for 40 cycles. The ΔCT value was calculated, using cel-miR-39 as the control gene, as the difference between the target gene and cel-miR-39, as follows: ΔΔCT = [(CT_target−gene_-CT_cel−miR−39_) Experimental group] – [(CT_target gene_-CT_cel−miR−39_) Control group]. Therefore, 2^−ΔΔCT^ represented the relative miRNA expression of the experimental and control groups.

### Statistical analysis

All data analysed in this study were processed using the SPSS software (version 22.0; IBM Corporation, Armonk, NY, USA). The distribution of continuous variables was evaluated using the Kolmogorov-Smirnov normality test. The results are presented as “mean ± standard deviation” for normally distributed data, and an independent sample t-test was used to compare the groups. Non-normally distributed data are presented as median (interquartile range), and nonparametric tests were used to analyse these data. For categorical variables, the chi-squared test was used. Logistic regression analysis was employed to determine the predictive power of clinical data for NOAF after AMI. The diagnostic efficacy of clinical indicators of NOAF after AMI was analysed using the receiver operating characteristic (ROC) curve and the area under the curve (AUC). Correlations among the variables in patients with NOAF were analysed using Spearman’s rank correlation. All statistical tests were two-tailed, and statistical significance was set at a P-value < 0.05.

## Results

### Basic data of the selected population

A total of 172 patients were included in this study, with 97 and 32 in the non-NOAF and NOAF groups, respectively. There were no significant differences between the groups regarding age, sex, hypertension, diabetes, total white blood cell count upon admission, neutrophil ratio, glomerular filtration rate (GFR), and levels of uric acid, brain natriuretic peptide (BNP), creatinine, Mg2+, and K+ (P > 0.05). The proportion of patients with a Glasgow Prognostic Score of 0 points was significantly higher than those with a score of 1 point (P < 0.01), and the incidence of Glasgow Prognostic Score elevation was higher in the NOAF group compared with the non-NOAF group (P < 0.05). The erythrocyte sedimentation rate was higher in the NOAF group than in the non-NOAF group (P < 0.05). Furthermore, the increase in left atrial diameter was significantly greater in the NOAF group compared with the non-NOAF group (P < 0.01; Table 1).

**Table 1:** Basic population and laboratory data on admission

Project		Non-NOAF group (N = 140)	NOAF group (N = 32)	χ^2^/t	P
Age (years)		61.18 ± 12.61	65.72 ± 13.01	-1.82	0.07
Males (%)		115 (82.1)	22 (68.8)	2.88	0.09
Diabetes mellitus (%)		37 (26.40)	6 (18.80)	0.82	0.37
Hypertension (%)		75 (53.60)	17 (53.10)	0.002	0.94
WBC (×10^9^/L)		10.8 (7.84–13.32)	10.72 (7.78–14.33)	-0.06	0.95
NEUT%		79.4 (70.23–85.95)	80.95 (68.75–85.63)	-0.3	0.77
Purine trione		386.56 ± 104.52	408.97 ± 109.62	-1.09	0.28
BNP		479 (148.25–1756)	747 (209.08–3346.50)	-1.27	0.2
Cr		73.3 (61.35–91.8)	72.6 (63.18–93.30)	-0.18	0.86
GFR		73.23 ± 21.13	68.57 ± 26.54	1.07	0.29
Mg^2+^		0.94 (0.89–1.025)	0.94 (0.87–0.10)	-0.48	0.63
K^+^		3.76 (3.53–4)	3.79 (3.46–4.24)	-0.32	0.75
The internal diameter of the left atrium		33 (31–36)	37.5 (33–39.75)	-2.9	0.01**
Glasgow prognostic score	0 [Number (%)]	104 (74.30)	16 (50)	6.94^a^	0.01**^a^
	1 [Number (%)]	34 (24.30)	15 (46.90)	0.01^b^	0.92^b^
	2 [Number (%)]	2 (1.40)	1 (3.10)	0.98^c^	0.32^c^
ESR (Normal: 0–38 mm/h)	Normal [Number (%)]	134 (95.7)	27 (84.4)	5.6	0.02*
	Elevated [Number (%)]	6 (4.3)	5 (15.6)		
hs-CRP (Normal: 0–5 mg/L)	Normal [Number (%)]	89 (63.6)	16 (50)	2.02	0.16
	Elevated [Number (%)]	51 (36.4)	16 (50)		

Note: NOAF, new-onset atrial fibrillation; WBC, white blood cell count; NEUT%, percentage of neutrophils; BNP, brain natriuretic peptide; Cr, creatinine; GFR, glomerular filtration rate; Mg^2+^, magnesium ion; K^+^ potassium ion; ESR, erythrocyte sedimentation rate; hs-CRP, high-sensitivity C-reactive protein. ^a^Glasgow prognostic score: 0 versus 1; ^b^Glasgow prognostic score: 1 point compared with 2 points; ^c^Glasgow prognostic score: 0 to 2; *P < 0.05; **P < 0.01

### Coronary artery lesion data

The enrolled patients diagnosed with myocardial infarction based on ECG examination were divided into two groups: the anterior wall group (including anterior, extensive anterior, lateral, lower, and posterior walls) and the non-ST elevation myocardial infarction (NSTEMI) group. The non-NOAF group had more NSTEMI infarctions compared with the NOAF group. Further analysis showed no significant difference between the number of patients with anterior and lower wall infarctions and those with NSTEMI (P > 0.05). Among patients with NOAF and non-NOAF, the number of single-, double-, and triple-branched coronary lesions was also not significantly different. (P > 0.05; Table 2).

**Table 2:** Number of coronary lesions, main culprit vessels, and infarction sites

Variable		NOAF		χ^2^	P-value
		No	Yes		
Number of coronary lesions	Single-branch lesion	29 (20.7)	3 (9.40)	2.27	0.32
	Double-branch lesions	32 (22.90)	9 (28.10)		
	Three-branch lesions	79 (56.40)	20 (62.50)		
Main culprit blood vessel	Left coronary circumflex branch	29 (20.7)	3 (9.4)	2.59	0.27
	Anterior descending	32 (22.90)	10 (31.30)		
	Right coronary artery	79 (56.40)	19 (59.40)		
Infarction site	Lower wall	39 (27.9)	14 (43.80)	0.29^a^	0.06^a^
	Anterior wall	46 (32.90)	13 (40.60)	4.35^b^	0.037^b^
	NSTEMI	55 (39.3)	5 (15.60)	6.68^c^	0.01*^c^

^a^lower wall versus anterior wall; ^b^anterior wall versus NSTEMI; ^c^lower wall versus NSTEMI; *P < 0.05; Data are presented as number (%)

NOAF, new-onset atrial fibrillation; NSTEMI, non-ST elevation myocardial infarction

### Multivariate correlation analysis

The inclusion of NOAF-related risk factors in the logistic regression model showed that the Glasgow prognostic score (odds ratio [OR], 2.375, 95% confidence interval [CI], 1.091–5.171), left atrial diameter (OR, 1.065; 95% CI, 1.011–1.122), and infarct size (OR, 0.778; 95% CI, 0.625–0.967) were independent risk factors for NOAF (Table 3).

**Table 3:** Multivariate logistic regression analysis of NOAF

Variable	B	Wald	OR	P-value	95% CI	
					Lower limit	Upper limit
ESR	0.845	1.43	2.327	0.232	0.583	9.293
Glasgow prognostic score	0.865	4.746	2.375	0.029	1.091	5.171
The internal diameter of the left atrium	0.063	5.549	1.065	0.018	1.011	1.122
Infarct site	-0.251	5.11	0.778	0.024	0.625	0.967

NOAF, new-onset atrial fibrillation; OR, odds ratio; CI, confidence interval; ESR, erythrocyte sedimentation rate

### ROC curve

**Fig. 1:**
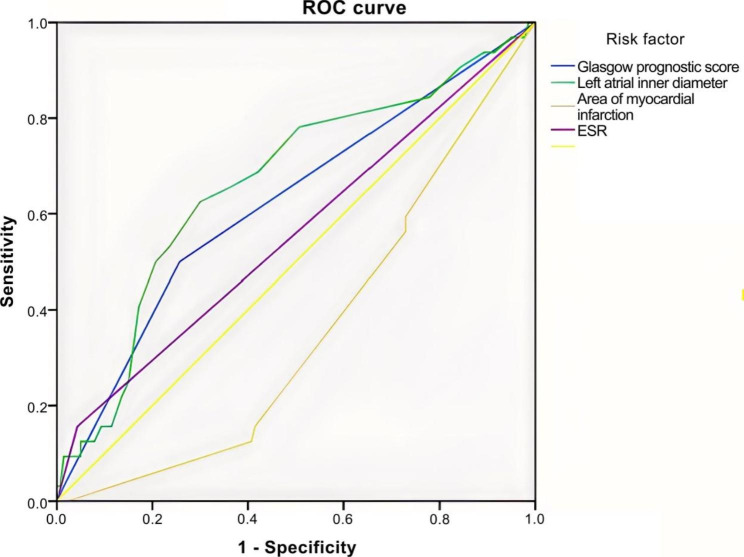
The ROC curve of related risk factors for new-onset atrial fibrillation after acute myocardial infarction prediction. ROC, receiver operating characteristic; ESR, erythrocyte sedimentation rate

The Glasgow prognostic score demonstrated an AUC of 0.62 in predicting NOAF. When using a cut-off value of 0.5 for the Glasgow prognostic score, the sensitivity and specificity for predicting NOAF were 50% and 74.3%, respectively. Regarding the left atrial diameter, the AUC was 0.66. When using a cut-off value of 35.5 mm for the internal diameter of the left atrium, the sensitivity and specificity for predicting NOAF were 62.5% and 70%, respectively (Fig. 1; Tables 4 and 5).

**Table 4:** AUC and 95% CI for NOAF occurrence predicted by independent risk factors

Variable	AUC	Standard error	P-value	95% CI	
				Lower limit	Upper limit
Glasgow prognostic score	0.62	0.06	0.03	0.51	0.734
Left atrial diameter	0.66	0.06	0.00	0.56	0.772
Infarct site	0.37	0.05	0.02	0.27	0.467

AUC, area under the curve; CI, confidence interval; NOAF, new-onset atrial fibrillation

**Table 5:** Specificity and sensitivity of independent risk factors for predicting NOAF

Variable	Cut-off value	Sensitivity	Specificity
Glasgow prognostic score	0.5	0.5	0.743
The internal diameter of the left atrium	35.5	0.625	0.7

### Quantitative plasma miR1 and miR133a expression

The plasma expression levels of miR1 and miR133a were determined using qRT-PCR. The normality of the miR1 and miR133a values was evaluated using the Shapiro-Wilk test. The nonparametric Mann-Whitney test was then used to compare the expression levels between the NOAF and non-NOAF groups. The Mann-Whitney test results indicated significantly higher expression levels of miR1 and miR133a in the NOAF group than in the non-NOAF group (P < 0.01; Table 6).

**Table 6:** Expression of miR1 and miR133a preoperatively and 1-day postoperatively

Variable	Non-NOAF (N = 97)	NOAF (N = 32)	P-value
miR1D0	1.00 (0.26–3.02)	22.00 (0.49–8715.79)	< 0.001
miR1D1	0.69 (0.26–1.79)	10.01 (0.44–10397.86)	< 0.001
miR133aD0	0.60 (0.16–2.83)	164.32 (0.54–20836.49)	< 0.001
miR133aD1	0.82(0.14–3.00)	33.61 (1.51–16278.85)	< 0.001

miR1D0, Preoperative miR1; miR1D1, Postoperative day 1 miR1; miR133aD0, preoperative miR133a; miR133aD1, miR133a on the first postoperative day

### Correlation between the expression of miR1 and miR133a preoperatively and 1-day postoperatively and clinical indicators

The relationship between the expression of miR1 and miR133a and several clinical indicators was analysed using the Spearman statistical test. The left atrial diameter was moderately related to the preoperative expression of miR1 (r = 0.163); the miR133a expression on the first day after surgery was associated with the percentage of neutrophils (r = 0.205), which were significant overall (r = 0.264; Table 7).

**Table 7:** Correlations between the expression of miR1 and miR133a preoperatively and 1-day postoperatively and clinical indicators

	Variable		miR1D0	miR1D1	miR133aD0	miR133aD1
Spearman correlation coefficient	ESR	r	0.024	0.006	0.002	0.023
		p	0.756	0.942	0.977	0.761
	Glasgow prognostic score	r	-0.029	-0.015	0.016	0.03
		p	0.704	0.85	0.84	0.697
	The internal diameter of the left atrium (mm)	r	0.163*	0.134	0.061	0.101
		p	0.032	0.08	0.429	0.187
	Infarct size	r	-0.14	-0.098	-0.17	-0.015
		p	-0.149	-0.104	0.054	0.193
	Percentage of neutrophils	r	0.148	0.125	0.205**	0.229**
		p	0.053	0.102	0.007	0.003

*The correlation was significant at p < 0.05 (two-tailed); **The correlation was significant at p < 0.01 (two-tailed). miR1D0, Preoperative miR1; miR1D1, Postoperative day 1 miR1; miR133aD0, preoperative miR133a; miR133aD1, miR133a on the first postoperative day; ESR, erythrocyte sedimentation rate

## Discussion

### Inflammatory factors and NOAF

Inflammation is associated with structural and electrical remodelling in the atrium and the occurrence and persistence of atrial fibrillation (AF) [18, 19]. Infections, including their type, duration, and severity, may impact atrial remodelling [20]. Multicentre studies [21, 22] have found an increased proportion of AF in patients with sepsis, indicating that pro-inflammatory and inflammatory mediators of blood circulation are associated with AF development [23]. After acute myocardial infarction (AMI), the inflammatory response is vital in myocardial remodelling [23, 24]. The Glasgow Prognostic Score (GPS) assesses serum albumin and C-reactive protein levels [25]. A drop in serum albumin level indicates a severe inflammatory response in the patient [26]. Therefore, the GPS can accurately reflect the degree of the inflammatory response. In our study, after excluding severe infectious diseases, we found that new-onset AF (NOAF) was related to the GPS and increased blood sedimentation. This suggests that NOAF after AMI is associated with inflammatory mechanisms.

### Infarct site and NOAF

Atrial ischemia or infarction can lead to electrical and structural remodelling of the atrium [27, 28]. Relevant studies suggest that ischemia can disrupt diastolic cytoplasmic Ca2 + flow. Notably, several factors, such as increased intracellular acidification and dephosphorylation of junction proteins, can result in local conduction blocks and facilitate the occurrence of atrial fibrillation [29, 30]. Furthermore, other studies indicate that proximal occlusion of the left spiral artery can cause reduced branch circulation in the atrioventricular node, contributing to the development of atrial fibrillation [31, 32]. In addition, a study found that coronary lesions originating from either the left or right coronary artery system could promote atrial fibrillation due to atrial ischemia [33]. In our study, patients with inferior and anterior wall infarctions were more likely to develop atrial fibrillation than those with non-ST-segment elevation myocardial infarction (NSTEMI). However, this association was not observed with diseased vessels, suggesting that the severity of ischemia is linked to new-onset atrial fibrillation (NOAF).

### miR1, miR133a, and NOAF

Injured cardiomyocytes after AMI promote inflammatory responses by releasing miR-1 and increasing the number of monocytes in the blood [34]. The increase in miR-1 can regulate atrial specificity, improve heart conduction, repolarisation, and heart rate, and reduce atrial fibrillation through the double-pore domain potassium channel TASK-1. TASK-1 is a weak inwardly rectifying acid-sensitive K + channel encoded by KCNK3 [35–37]. Yang et al. [38] found that miR-1 overexpression can be inhibited by KCNJ2, which encodes the K + channel subunit Kir2.1, and GJA1, which encodes connexin-43. This inhibition slows the conduction and depolarisation of the cytoplasmic membrane and inhibits atrial fibrillation. In patients with persistent atrial fibrillation, miR-1 expression decreases, leading to increased inward rectifier current activity [39]. Yuan et al. [40] found that miR-1 levels were significantly lower in geriatric atrial fibrillation patients compared with non-atrial fibrillation patients. However, Terentyev et al. reported that increased miR-1 levels in cardiomyocytes lead to selectively decreased expression of the B56α regulatory subunit of protein phosphatase 2 A. This reduction causes reduced medium-mediated dephosphorylation of the L-type calcium channel (LTCC) and ryanodine receptor 2 (RyR2), resulting in increased calcium/calmodulin-dependent kinase II phosphorylation of LTCC and RyR2. Consequently, an inward endoplasmic reticulum Ca2 + current is induced, promoting arrhythmia development [41]. Wiedmann et al. [42] found that miR-1 was associated with the atrial collagen alpha-2(I) chain, and pro-apoptotic miR-1 was increased in the right atrial tissue of patients with NOAF after coronary atrial bypass graft compared with patients without atrial fibrillation. In this study, we observed that miR-1 was elevated in NOAF after AMI and was associated with increased atrial diameter, suggesting that increased miR-1 levels in patients were associated with myocardial structural remodelling, leading to atrial fibrillation. In addition, miR-133a levels were significantly higher before surgery and on the first day after surgery in the NOAF group compared with the non-NOAF group. Zhu et al. [43] discovered that miR-133a can activate macrophage migration inhibitory factors, resulting in increased Akt phosphorylation and Bcl-2 expression, and decreased caspase-3 expression, fibrosis, and apoptosis. Similarly, miR-133a can also promote the expression of vascular endothelial growth factor protein in human umbilical cord venous blood to reduce the occurrence of atrial fibrillation. Related studies suggested that miRNA-133a may be involved in downregulating CD47 in human HeLa cancer cells [44]. CD47 can inhibit the migration and adhesion of neutrophils [45]. Through a 1-year follow-up, research has shown that activating the miR-33/SIRT1 pathway increases inflammation and coagulation processes, thrombus burden, and the formation of distant embolisms [46]. In our analysis, elevated miR-133a levels were positively associated with the neutrophil percentage, inhibiting the inflammatory response. miR-133a reduced the occurrence of NOAF by stabilising myocardial structural remodelling and inhibiting the inflammatory response.

Overall, the Glasgow Prognostic Score and left atrial diameter were independent risk factors for NOAF after AMI. The mechanism associated with this condition may involve elevated miR-1 levels and an enlarged left atrial diameter. Conversely, miR-133a is protective in inhibiting the inflammatory response in cardiomyocytes. Administering non-vitamin K antagonist oral anticoagulants (NOACs) for anticoagulation therapy in patients with atrial fibrillation is recommended. In the AFTER-2 study conducted in Turkey, patients with high time in therapeutic range (TTR) on warfarin treatment showed no significant difference in the incidence of ischemic cerebrovascular disease/transient ischemic attacks (CVD/TIA), intracranial haemorrhage, and mortality as primary outcomes [47, 48]. Increasing the frequency of follow-up visits and minimising adverse events is recommended for patients with new-onset atrial fibrillation after acute myocardial infarction.

This was a single-centre retrospective study with a small sample size. Multicentre studies with large sample sizes are required to validate our findings. In addition, due to the short duration or delayed observation of atrial fibrillation, the results of previously asymptomatic patients with atrial fibrillation, and the influence of potential immune-related diseases on miRNA expression, could not be excluded entirely. Finally, the lack of further analysis of the onset of NOAF and miRNA changes in patients with AMI may have been a limitation of this study.

## Conclusions

In this study, we identified a mechanism associated with NOAF after AMI that involves an inflammatory response and changes in miRNA expression. Specifically, we observed a detrimental role of miR-1 associated with an increased left atrial diameter. Conversely, miR-133a may play a protective role in NOAF by increasing the neutrophil ratio, thereby reducing the inflammatory response. These findings provide novel insights into the mechanism of NOAF after AMI, which could contribute to developing innovative therapeutic strategies for this condition.

## Data Availability

The datasets and materials used and/or analysed during the current study are available from the corresponding author upon reasonable request.

## Abbreviations

AMI: Acute myocardial infarction
AUC: Area under the curve
BNP: Brain natriuretic peptide
CI: Confidence interval
ECG: Electrocardiogram
GFR: Glomerular filtration rate
LTCC: L-type calcium channel
NOAF: New-onset atrial fibrillation
NSTEMI: Non-ST elevation myocardial infarction
OR: Odds ratio
PCI: Percutaneous coronary intervention
qRT-PCR: Quantitative real-time polymerase chain reaction
ROC: Receiver operating characteristic
RyR2: Ryanodine receptor 2
